# Performance of a semiconductor SPECT system: comparison with a conventional Anger-type SPECT instrument

**DOI:** 10.1007/s12149-012-0653-9

**Published:** 2012-09-06

**Authors:** Yasuyuki Takahashi, Masao Miyagawa, Yoshiko Nishiyama, Hayato Ishimura, Teruhito Mochizuki

**Affiliations:** 1 Department of Nuclear Medicine Technology, Gunma Prefectural College of Health Sciences, 323-1 Kamioki-cho, Maebashi, 371-0052 Japan; 2 Department of Radiology, Ehime University Graduate School of Medicine, Toon, Japan; 3 Department of Radiological Technology, Ehime University Hospital, Toon, Japan

**Keywords:** Semiconductor camera, Spatial resolution, Sensitivity, Myocardial SPECT

## Abstract

**Objective:**

The performance of a new single photon emission computed tomography (SPECT) scanner with a cadmium-zinc-telluride (CZT) solid-state semiconductor detector (Discovery NM 530c; D530c) was evaluated and compared to a conventional Anger-type SPECT with a dual-detector camera (Infinia).

**Methods:**

Three different phantom studies were performed. Full width at half maximum (FWHM) was measured using line sources placed at different locations in a cylindrical phantom. Uniformity was measured using cylindrical phantoms with 3 different diameters (80, 120, and 160 mm). Spatial resolution was evaluated using hot-rod phantoms of various diameters (5, 9, 13, 16, and 20 mm). Three different myocardial phantom studies were also performed, acquiring projection data with and without defects, and evaluating the interference of liver and gallbladder radioactivity. In a clinical study, the D530c employed list-mode raw data acquisition with electrocardiogram (ECG)-gated acquisition over a 10-min period. From the 10-min projection data, 1-, 3-, 5-, 7- and 10-min SPECT images were reconstructed.

**Results:**

The FWHM of the D503c was 1.73–3.48 mm (without water) and 3.88–6.64 mm (with water), whereas the FWHM of the Infinia was 8.17–12.63 mm (without water) and 15.48–16.28 mm (with water). Non-uniformity was larger for the D530c than for the Infinia. Truncation artifacts were also observed with the D530c in a Φ160 mm phantom. The contrast ratio, as defined by myocardial defect/non-defect ratio, was better for the D530c than for the Infinia, and the influence from liver and gallbladder radioactivities was less. Quantitative gated SPECT (QGS) software demonstrated significant differences between data captured over a 10-min period, relative to those acquired over periods of <5 min; there was no difference between ejection fractions calculated using data capture for periods ≥5 min (*p* < 0.05).

**Conclusions:**

The D530c is superior to the Infinia, with regard to both spatial resolution and sensitivity. In this study, these advantages were confirmed by the myocardial phantom and in a clinical setting, using the QGS software.

## Introduction

A recently released semiconductor detector for single photon emission computed tomography (SPECT) offers several advantages over the conventional Anger-type SPECT. Instruments of both types were compared in this evaluation, in conjunction with the necessary software, to perform quantitative gated SPECT (QGS) [1]. The guidelines recommend a 20–30 min acquisition time for conventional systems [2, 3]. However, the ability of the new SPECTs to convert absorbed gamma rays directly to an electric charge allows for enhanced sensitivity and spatial resolution [4–6]. Therefore, myocardial SPECT can be acquired within as little as 3–6 min [4]. The data acquisition method of the new SPECT scanner differs from that of conventional SPECT scanners in that it has a smaller field of view (FOV). We evaluated the spatial resolution and sensitivity (acquisition time for QGS) of a new SPECT scanner relative to a conventional instrument.

## Materials and methods

### Instruments

The performances of 2 SPECT scanners were evaluated and compared in this study. The first was a new SPECT scanner with a cadmium-zinc-telluride (CZT) solid-state semiconductor detector (Discovery NM 530c; D530c, GE Healthcare, Milwaukee, WI, USA). A conventional Anger-type SPECT scanner with a NaI(Tl) scintillation crystal and a dual-detector camera (Infinia; GE Healthcare) was similarly evaluated. QGS was performed using the output of both instruments using software developed at the Cedars Sinai Medical Center (Los Angeles, CA, USA) [1].

The D530c, equipped with 19 pinhole collimators, employed list-mode raw data acquisition over a 10-min interval. The three-dimensional volume (quality field-of view) for high quality imaging was contained within a sphere, approximately 19 cm in diameter [6]. The matrix size was 70 × 70, and the image reconstruction voxel size was 4.0 × 4.0 × 4.0 mm. The instrument was equipped with a Xeleris data processor (GE Healthcare). The phantom studies were conducted over an imaging period of 5 min. In the clinical study, QGS data were acquired over 10 min. From the 10-min projection data, 1-, 3-, 5-, 7-, and 10-min SPECT images were reconstructed.

The Infinia is a dual-detector, gamma camera system equipped with low-energy, high-resolution collimators. The matrix size for this instrument was 64 × 64, having a reconstructed pixel size of 6.8 × 6.8 × 6.8 mm. A circular rotation, with a step-and-shoot mode (30 s/projection data), was applied. Cylinder phantoms were scanned at 3° intervals over 360° using the step-and-shoot mode. The myocardial phantom was scanned at 6° intervals over 360° (20 s/step, 20 min in total). Patients were scanned with electrocardiogram (ECG)-gating at 6° intervals over 360° (40 s/step, 20 min in total) in a supine position.

In the line-source, hot-rod, and cylinder-phantom studies, the D530c data were reconstructed using maximum likelihood expectation maximization (ML-EM) with 250 iterations [6]. Reconstruction of the Infinia data used 50 iterations [7]. In the myocardial phantom study, reconstruction with the D530c was based on the implementation of a 3D iterative Bayesian reconstruction algorithm [8]. Reconstruction with the Infinia system used ordered subsets-EM (OS-EM) [7]. For the OS-EM parameter, iteration number 10 and 5 subsets were used. For all reconstructions, a Butterworth filter (order 7, cutoff frequency = 0.37 cycles/cm) [5] was used as a post-filter.

#### Spatial resolution

**Fig. 1:**
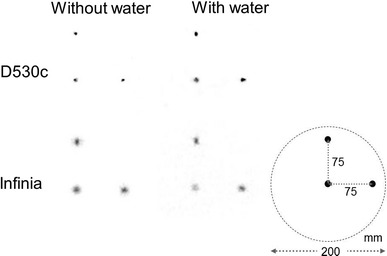
A single photon emission computed tomography image of the line-source phantom. The line sources were set in the cylinder with and without water (scatter)

Full width at half maximum (FWHM) was measured using 3 ^99m^Tc line sources those were placed in a cylindrical phantom, 200 mm in diameter and 200 mm in length. The diameter of the 3 line sources was 1.0 mm and they were filled with 74 MBq ^99m^Tc. The interval between the lines was 75 mm (Fig. 1, lower right). Data were acquired with and without water (scatter) in the cylinder. FWHM values of the line sources in the central, radial, and tangential directions were generated [9].

#### Uniformity

Phantom studies were performed using cylindrical phantoms with diameters of 80, 120, and 160 mm, each uniformly filled with ^99m^Tc. The acquisition time was set so as to record 100 kilocounts per frame. The degree of uniformity over each cylindrical phantom was evaluated by employing profile curves along the *Y*-axis in the transverse plane. SPECT values of the profile curves were normalized to the maximum SPECT counts in each profile curve. The average ± 2 standard deviations of normalized SPECT values (%) were considered uniform.

#### Hot-rod phantom

**Fig. 2:**
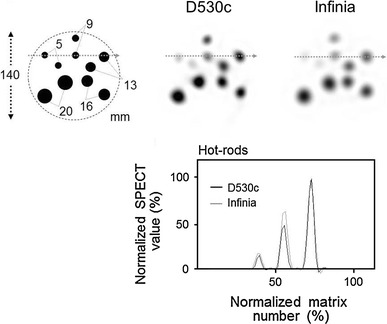
A single photon emission computed tomography image from a uniformity phantom with a diameter of 80, 120, and 160 mm

Hot-rods were set in a cylindrical phantom, 140 mm in diameter. The hot-rod diameters were 5, 9, 13, 16, and 20 mm and their locations are shown in Fig. 2, upper left. The cylinder was not filled with a water background. Transverse-plane profile curves that passed through the centers of the rod cross sections were generated and evaluated.

#### Myocardial phantom

The myocardial phantom study was conducted using an anthropomorphic phantom (HL-D PH-25; Kyoto-Kagaku, Kyoto, Japan). Radioactive medium (88.5 kBq/mL) was injected into the area representing the myocardium. Three different phantom conditions were evaluated: (a) without a defect in the absence of radioactivity in the liver or gallbladder; (b) the presence of a defect but without radioactivity in the liver or gallbladder; and (c) without a defect in the presence of radioactivity in the liver and gallbladder. In condition (b), a 20-mm defect was set in the anterior wall. In condition (c), radioactivity was established in the myocardium, liver, and gallbladder in the ratio of 1:1:2. The resultant images were compared using an inferoposterior wall to anterior wall contrast ratio.

#### Human study

Twenty patients with coronary artery disease (17 men and 3 women; mean age 66.2 ± 9.7 years; range 42–70 years) were studied. In each subject, 259 MBq of ^99m^Tc-tetrofosmin was injected intravenously, under stress; data acquisition was initiated 20 min after injection [1]. SPECT images were first acquired with the D530c (for 10 min) and then with the Infinia (for 20 min). From the projection data, 1-, 3-, 5-, 7-, and 10-min D530c images were reconstructed, and the ejection fraction (EF) was calculated using the QGS software for each reconstruction time.

### Statistical analysis

Values are described as means ± standard deviations (SD). Data were analyzed with JMP 9.0 software (SAS Institute Japan, Tokyo, Japan). One-way repeated-measures analysis of variance (ANOVA), using the least-square differences post hoc test, was used to compare the 5 different acquisition times for processing by QGS software. Probability values <0.05 were considered statistically significant.

## Results

### Spatial resolution

The position of the ^99m^Tc line sources and the reconstructed images with and without scatter (water) are shown in Fig. 1. The measured FWHM values are summarized in Table 1. The D530c system provided better FWHM values under all conditions.

**Table 1:** FWHM by three ^99m^Tc line sources

	Without water	With water
D530c		
Central	3.00	6.64
Tangential	3.48	5.03
Radial	1.73	3.88
Infinia		
Central	11.05	15.48
Tangential	12.63	16.28
Radial	8.17	15.61

Unit: mm

### Uniformity

**Fig. 3:**
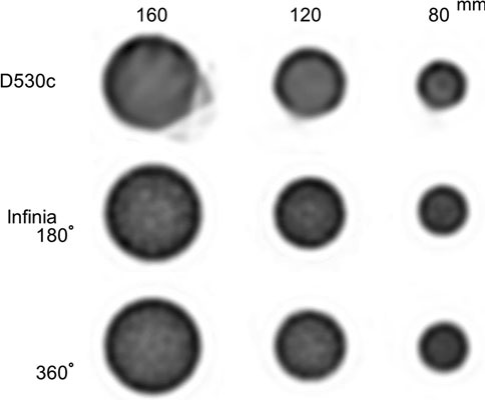
Positioning of the hot-rod phantom. Single photon emission computed tomography images of the hot-rod phantoms with diameters of 5, 9, 13, 16, and 20 mm are shown. The lower portion illustrates the profile curve of the *dotted line* shown in the illustration

The SPECT images of the cylinder phantoms are shown in Fig. 3. Truncation artifacts were only observed at the 3 o’clock position with the D530c, using the Φ160 mm phantom. The profile curve was used to estimate the uniformity along the *Y*-axis of the cylinder phantom. When the SPECT count at the center was divided by the average counts on both edges of the 80 and 120 mm phantom using profile curve analyses, the ratios were 76.2 ± 3.9 and 67.9 ± 4.5 % for the D530c, 85.9 ± 5.5 and 77.9 ± 8.4 % for the Infinia at 180°, and 84.8 ± 2.9, 75.1 ± 4.5 % for the Infinia at 360°.

#### Hot-rods phantom

The positioning of the hot-rods is illustrated in Fig. 2 (upper left). Blurring of the edges of the hot-rods was more prominent for the images produced by the Infinia instrument (Fig. 2, upper middle and right). In other words, the edges of the hot-rods were represented more sharply by the D530c. The profile curves of the D530c also showed sharper edges, i.e., a narrower width (Fig. 2, lower).

### Myocardial phantom

**Fig. 4:**
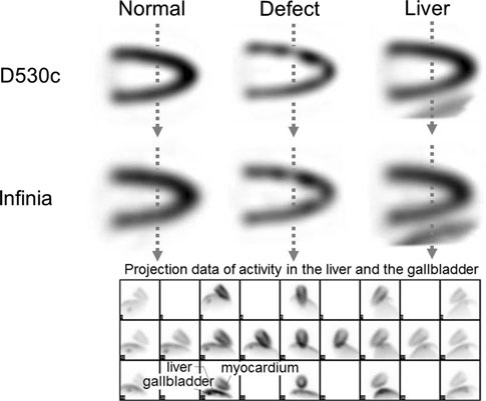
Horizontal single photon emission computed tomography images of a myocardial phantom. The left image is normal (no defect); a 20 mm defect is set in the anterior in the center image; and the radioactivity levels of the liver and gallbladder were set to simulate the human body in the right images (no defect)

**Fig. 5:**
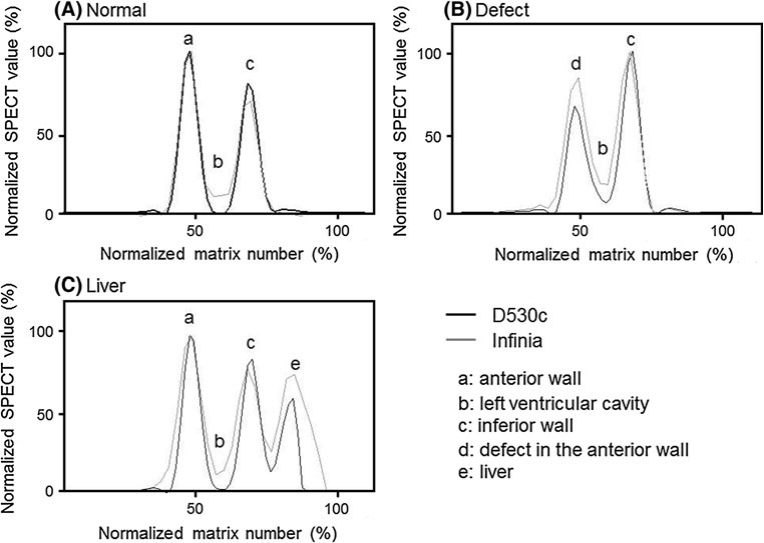
Profile curve analysis at the dotted arrow in each position. **a** Normal myocardial phantom. **b** Myocardial phantom including a defect. **c** Myocardial phantom including the liver and gallbladder

Images of the myocardial phantom under the 3 conditions (no defect, defect, and no defect with liver and gallbladder radioactivity) are shown in Fig. 4. Arrows show the position of the profile curve (Fig. 4, upper and middle). Projection images (no defect with liver and gallbladder radioactivity) of the 19 pinhole detectors are shown in Fig. 4 (lower row). The profile curves corresponding to Fig. 4 are shown in Fig. 5.

The ratios of the inferior wall count divided by the anterior count were 80.4 % for the D530c and 70.3 % for the Infinia. The ratios of the anterior defect count divided by the inferior wall count were 66.5 % for the D530c and 84.7 % for the Infinia (d in Fig. 5b). The profile curves indicate that the D530c depicted the small defect more clearly than did the Infinia.

The ratios of the inferior wall count divided by the anterior wall count with liver and gallbladder radioactivity were 84.1 % for the D530c and 80.5 % for the Infinia. The difference in the ratios of the inferior wall count divided by the anterior between the D530c and the Infinia was smaller when the liver and gallbladder signals were present.

### Human study

**Fig. 6:**
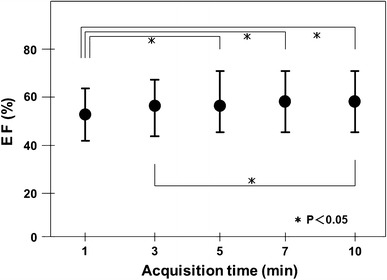
Ejection fraction calculated using 1-, 3-, 5-, 7-, and 10-min data in 20 cases

The EF values obtained by QGS software with acquisition times of 1, 3, 5, 7, and 10 min are shown in Fig. 6. The EF value obtained following a 1-min acquisition was significantly different from those calculated after acquisition times of 5, 7, and 10 min (*p* < 0.05). The EF value of the 3-min acquisition was also different from that of the 10-min acquisition (*p* < 0.05). However, there were no significant differences among the EF values obtained following acquisition times of 5, 7, and 10 min.

## Discussion

Before the advent of the D530c used in this study, a different type of semiconductor (CZT) detector SPECT scanner was developed as the D-SPECT [8]. The D-SPECT was superior to conventional SPECT scanners with an NaI(Tl) scintillation crystal in terms of its sensitivity and energy resolution. Although both the D-SPECT and the D530c scanners are equipped with semiconductor detectors, there are several differences: the solid state detector unit of D-SPECT rotates inside a small gantry, whereas that of the D530c does not rotate; the D-SPECT consists of 9 arrays of CZT detectors, whereas the D530c has 19 pinhole collimators. As well as higher sensitivity and better energy resolution, higher spatial resolution can be expected from the use of the pinhole collimators and a 3D iterative Bayesian reconstruction algorithm [10]. Therefore, the performance of the newly developed D530c SPECT scanner was investigated, mainly through phantom studies, and compared to results obtained using a conventional Anger type (Infinia) dual-head SPECT scanner.

In the line-source phantom study, the FWHM of the D530c was superior to that of the Infinia, indicating higher spatial resolution. Hot-rods and myocardial phantom studies also indicated better spatial resolution with the D530c. Higher spatial resolution implies higher diagnostic accuracy for myocardial ischemia [6], but this will need to be confirmed in future clinical studies. For other conditions, a low-dose, single-day stress–rest protocol performed using D530c has been reported to provide good sensitivity and specificity in detecting coronary artery disease [11].

In the cylindrical phantom study, counts at the center of the cylinder tended to be lower when measured by the D530c than by the Infinia. Although the influence of non-uniformity on the clinical imaging of the myocardium is unclear, this is an important finding. Non-uniformity may be derived during 180° data acquisition, and since truncation artifacts may occur in larger subjects, careful positioning of the D530c is necessary, particularly for large hearts.

The myocardial phantom study demonstrated that the counts in the inferior wall were lower than in the other walls. Optimized attenuation correction might be necessary to improve the wall uniformity and to acquire better diagnostic performance, as in the case of the Discovery NM/CT 570c [12]. The influence of high levels of radioactivity in the liver and gallbladder on the inferior myocardial wall was less with the D530c in this study. However, the location and volume of the high counts would be likely to affect the magnitude of this influence. There are many reports in which high activity in the liver and/or gallbladder caused imaging artifacts, i.e., lower counts in the inferior wall [13]. The influence of high radioactivity in the liver and gallbladder may be reduced by 180° data acquisition [14], but the D530c pinhole collimators located in the lower row are likely to detect the myocardium through the liver, which might cause lower counts in the inferior wall in the clinical image.

In the clinical study, EF values obtained by QGS stabilized within 5 min in the D530c study. The image quality was acceptable for the QGS software after a 3-min acquisition time in the D530c. The results of this study also confirmed that the accuracy of the EF at 5 min, or even at 3 min, was sufficient for the assessment of myocardial perfusion.

## Conclusions

The D530c CZT semiconductor, pinhole detector SPECT scanner had a higher spatial resolution than did the conventional NaI(Tl) Anger camera. The high sensitivity of the D530c allows for a shorter acquisition time, 5 min is sufficient for QGS in the clinical setting.
